# Comparative Study of Photoelectric Properties of Metamorphic InAs/InGaAs and InAs/GaAs Quantum Dot Structures

**DOI:** 10.1186/s11671-017-2091-z

**Published:** 2017-05-05

**Authors:** Sergii Golovynskyi, Luca Seravalli, Oleksandr Datsenko, Giovanna Trevisi, Paola Frigeri, Enos Gombia, Iuliia Golovynska, Serhiy V. Kondratenko, Junle Qu, Tymish Y. Ohulchanskyy

**Affiliations:** 1 College of Optoelectronic Engineering, Key Laboratory of Optoelectronic Devices and Systems of Ministry of Education and Guangdong Province, Shenzhen University, 518060 Shenzhen, People’s Republic of China; 2 Institute of Semiconductor Physics, NAS of Ukraine, 45 Nauki Ave, 03028 Kyiv, Ukraine; 3 Institute of Materials for Electronics and Magnetism, CNR-IMEM, Parco delle Scienze 37a, I-43100 Parma, Italy; 4 Department of Physics, Taras Shevchenko National University of Kyiv, 64 Volodymyrska St, 01601 Kyiv, Ukraine

**Keywords:** Nanostructure, Quantum dot, Metamorphic, InAs/InGaAs, Photoconductivity, Photoluminescence, Photovoltage, Absorption, Defects, Photoelectric

## Abstract

Optical and photoelectric properties of metamorphic InAs/InGaAs and conventional pseudomorphic InAs/GaAs quantum dot (QD) structures were studied. We used two different electrical contact configurations that allowed us to have the current flow (i) only through QDs and embedding layers and (ii) through all the structure, including the GaAs substrate (wafer). Different optical transitions between states of QDs, wetting layers, GaAs or InGaAs buffers, and defect-related centers were studied by means of photovoltage (PV), photoconductivity (PC), photoluminescence (PL), and absorption spectroscopies. It was shown that the use of the InGaAs buffer spectrally shifted the maximum of the QD PL band to 1.3 μm (telecommunication range) without a decrease in the yield. Photosensitivity for the metamorphic QDs was found to be higher than that in GaAs buffer while the photoresponses for both metamorphic and pseudomorphic buffer layers were similar. The mechanisms of PV and PC were discussed for both structures. The dissimilarities in properties of the studied structures are explained in terms of the different design. A critical influence of the defects on the photoelectrical properties of both structures was observed in the spectral range from 0.68 to 1.0 eV for contact configuration (ii), i.e., in the case of electrically active GaAs wafer. No effect of such defects on the photoelectric spectra was found for configuration (i), when the structures were contacted to the top and bottom buffers; only a 0.83 eV feature was observed in the photocurrent spectrum of pseudomorphic structure and interpreted to be related to defects close to InAs/GaAs QDs.

## Background

InAs/InGaAs metamorphic semiconductor quantum dots (QDs) are interesting nanostructures that allow to have emission in the 1.3 and 1.55 μm ranges from GaAs-based nanostructures [1–3]. InAs QDs embedded in InGaAs matrix provide specific features, such as lattice mismatch and band discontinuities between the QDs and confining layers, which can be very useful for an adroit engineering of properties. In particular, the growth of InAs on the InGaAs metamorphic buffers (MB) allows for reduction in the strain accumulated in QDs and, hence, modification of the energy of QD levels [4–7].

Recently, single photon emission at 1.3 μm from the metamorphic QDs has been demonstrated [8, 9], alongside the feasibility of photon emission evolution at such crucial wavelength and the perspective of a further extension of wavelength of emitted quantum light up to 1.55 μm [10]. Beside these very relevant results for quantum science applications, such nanostructures have been used for other novel devices such as metamorphic QD lasers [11, 12] and metamorphic QD solar cells [13]. Furthermore, a new design based on the metamorphic QDs for Broadband SLEDs (SuperLuminescent Diodes), devices of great interest for medical imaging applications, has been proposed. This design predicts a relevant increase in the bandwidth of operation, even at wavelengths as long as 1.6 μm [14]. The emission efficiency of metamorphic InAs/InGaAs QDs has been demonstrated to be as high as the one of standard InAs/GaAs QDs [4, 15]. Also, it was shown that InAs/InGaAs QDs were sensitive to normal incident light. This allows for the development of interband QD infrared photodetectors operating at lower energies than well-known InAs/GaAs ones [16–18].

While considerable research on the morphological and optical properties of the InAs/InGaAs QD nanostructures has been performed during last years [19–21], more work on the photoelectrical properties is still needed to provide a quantitative assessment of the role of strain-related defects in MBs and interfaces in device performances. A very relevant task is the suppression of the influence of the GaAs substrate and related interfaces on device performance, as they might be highly filled by defect traps and non-radiative recombination levels as well as dopant centers [22, 23]. Deep-level transient spectroscopy (DLTS) studies of InAs/In(Ga)As nanostructures [24, 25] give the major information on the defect level location within the bandgap, while the less-studied photoelectrical properties of the defects can be well determined by deep-level thermally stimulated conductivity (TSC) and photoconductivity (PC) spectroscopy at direct current [26–30]. PC studies give information about the defects as well as the transitions involving QDs, wetting layer (WL), InGaAs buffers and substrate, carrier transport, and other mechanisms of nonequilibrium processes [27], examining comprehensively the photoelectrical properties of the structure.

In a previous publication [30], we have carried out an in-depth study of a single metamorphic QD structure grown by molecular beam epitaxy (MBE) on semi-insulating (*si*)-GaAs substrates. We focused on the complex quantum-level system, composed by the QDs, WL, InGaAs embedding layer, and *n*-doped MB. It has been shown that the metamorphic QDs absorb and emit in the 1.3-μm range at room temperature (RT), while maintaining an efficient emission. The vertical configuration, used for photoelectrical characterization, consisted of two contacts deposited to MBE grown buffer layers that allowed to avoid the flow of electrical current through the *n*-InGaAs/*n*-GaAs and *n*-GaAs/substrate interfaces. We showed that by depositing a thick InGaAs MB on the substrate, we have achieved a strong suppression of the impact of the interface on the electrically active layers. While considerable advances in the understanding of the structure peculiarities were reached, a definitive assessment of the structure properties in comparison with the conventional InAs/GaAs QDs was not possible.

In this work, we compared the photoelectric properties of the metamorphic InAs/InGaAs QD structure with those of a standard InAs/GaAs QD one. The GaAs structure has been designed in order to have a direct comparison with the metamorphic one, providing a reference to ascertain the role of the metamorphic InGaAs layer on photoelectric properties. This allows to compare QD emission and photosensitivity from the two structures because of the less number of influencing parameters. The photoelectrical measurements are carried out at vertical configurations for electrical contacts: (i) to the MBE layers to characterize only QDs and embedding layers and (ii) to the top and substrate of the samples in order to evaluate the effect of GaAs/InGaAs and substrate/*n*-GaAs interfaces on the carrier photogeneration and transport.

## Methods

**Fig. 1:**
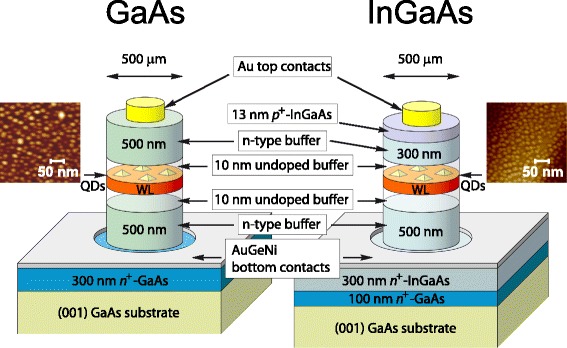
(*Color online*) Schematics of the metamorphic InAs/In_0.15_Ga_0.85_As/*si*-GaAs and InAs/GaAs/*si*-GaAs QD samples investigated: layer thickness, composition, and doping are indicated; AFM images of the uncapped structures are shown as well

The structures were prepared by MBE on (001) *si*-GaAs substrates. Substrates were provided by Wafer Technology that specified them to be *n*-type, with values of 3 × 10^7^ cm^−3^ carrier concentration, thickness of 500 μm, and a resistivity of 2 × 10^7^ Ω cm. The metamorphic InAs/InGaAs QD structure consists of (i) 0.1 μm *n* ^*+*^-GaAs buffer layer grown at 600 °C, (ii) 300 nm thick *n* ^*+*^-doped In_0.15_Ga_0.85_As MB with *n* = 5 × 10^18^ cm^−3^ grown at 490 °C, (iii) 500 nm thick *n*-doped In_0.15_Ga_0.85_As MB with *n* = 3 × 10^16^ cm^−3^ grown at 490 °C, (iv) 3.0 monolayers of self-assembled InAs QDs on a WL grown by atomic layer MBE (ALMBE) at 460 °C embedded in the middle of a 20 nm undoped In_0.15_Ga_0.85_As layer, (v) 300 nm *n*-doped In_0.15_Ga_0.85_As upper capping layer with *n* = 3 × 10^16^ cm^−3^ grown at 490 °C, and (vi) 13 nm *p* ^+^-doped In_0.15_Ga_0.85_As cap with *p* = 2 × 10^18^ cm^−3^ grown at 490 °C (Fig. 1) [4, 15]. As discussed in Ref. [30], undoped layers were used to separate QDs from *n*-doped regions to reduce the effect of non-radiative recombination centers, thus maximizing the QD light emission efficiency.

The InAs/GaAs QD structure consists of (i) 0.3 μm *n* ^*+*^-GaAs buffer layer with *n* = 5 × 10^18^ cm^−3^ grown at 600 °C, (ii) 500 nm thick *n*-doped GaAs MB with *n* = 3 × 10^16^ cm^−3^ grown at 600 °C, (iii) 3.0 monolayers of InAs QDs grown by ALMBE at 460 °C embedded in a 20 nm undoped GaAs layer, and (iv) 500 nm *n*-doped GaAs upper capping layer with *n* = 3 × 10^16^ cm^−3^ grown at 600 °C (Fig. 1). Atomic force microscopy (AFM) images of the uncapped structures are shown in Fig. 1. Previous analysis of AFM data provided QD densities of 1 × 10^11^ cm^-2^ and QD dimensions of 20 nm for base diameters and 4.9 nm for heights for metamorphic QDs [4, 31]. For standard QDs, surface densities are 2 × 10^11^ cm^−2^, base diameters are 21 nm, and heights are 5.0 nm [4, 32].

As shown in the Fig. 1, the vertical (in the growth direction) electrical contacts were made by defining circular mesas with 500 μm diameter and by etching of the *n*-InGaAs and *n*-GaAs buffers down to *n* ^*+*^-layer in a solution of C_6_H_8_O_7_:H_2_O:H_2_O_2_ (5:5:1) at 20 °C. Ohmic contacts were obtained by evaporating and annealing (at 400 °C for 1 min in nitrogen atmosphere) the Au_0.83_Ge_0.12_Ni_0.05_ alloy [33, 34] on the bottom *n* ^+^-InGaAs and *n* ^+^-GaAs layers, respectively. Au top contacts were then evaporated with a diameter of 400 μm and a thickness of 70 nm. Thick indium contacts were also soldered on the substrate bottoms of other pieces of same samples and annealed at 400 °C, in order to have measurements also with current flowing through the GaAs buffer and *si*-GaAs substrate. Wires were soldered to the contact layers of all four samples and connected to sample holder pins. It should be noted that the contacts of indium with *si*- and *n*-GaAs substrates are generally reported to be ohmic, not Schottky [35–37]. We have verified this by the IV measurements of the In-GaAs contacts; the current-voltage characteristics were found to be linear (data not shown).

The thin *p* ^+^-InGaAs layer between the Au contact and the *n*-InGaAs layer is used to enhance the barrier height since the structure obtained by the simple deposition of a metal on *n*-InGaAs exhibits a relatively low Schottky barrier height, following the approach proposed in Ref. [38] and already used in other works [39, 40]. An extended analysis of the electrical characteristics of metal/*p* ^+^-cap/InGaAs/InP structures is reported in Ref. [41], where the band bending profile of the structure is also shown. So the deposition of thin *p* ^+^-InGaAs layer provides approximate similarity of Schottky barrier height of top Au contact maintaining resemblance of barrier profile for both metamorphic and InAs/GaAs structures.

For structure and contact designing as well as understanding of the energy profile for both structures composed by the InGaAs or GaAs MBs, In(Ga)As QDs, undoped cap layer, and Au/AuGeNi contacts, the calculations were carried out using Tibercad software [42]. Band profiles were modeled in the drift-diffusion approximation, taking in consideration strain conditions, densities of traps related to defects at the InGaAs/GaAs interface region, depletion layers near contacts, and appropriate Schottky barriers heights. Calculating QD band offsets the data from AFM and photoluminescence (PL) measurements were utilized; the simulations were performed, considering experimentally obtained ground state transition energy (see the “Results” section), height and lateral width of QDs, and a low content of Ga in InAs QDs. However, the theoretical study of QDs is out of scope of this paper, and QD modeling has been performed only for representation of whole heterostructure band profiles between contacts.

The PL characterization was performed by exciting the sample with a laser light at 532 nm with power densities of 5 W/cm^2^ at 10 and 300 K; emission spectra were measured by a fast Fourier transform spectrometer with a cooled Ge detector.

Vertical photocurrent (photosensitivity) and photovoltage (PV) spectra were measured using an infrared prism spectrometer in the 0.6 to 1.8 eV range using normal incidence excitation geometry at RT (300 K) and were normalized by the intensity of light source. The photocurrent was measured using a current amplifier and direct current technique, with a bias voltage between 0 and −4 V. The current was measured with a Picotest M3511A multimeter as a voltage signal drop across a series load resistance of 1 kΩ (see the inset in Fig. 5): such signal was orders of magnitudes lower than the voltage drop across the sample. The current-voltage characteristics were measured in the dark and under illumination with intensity of 1.5 mW/cm^2^ at RT. The absorption spectrum was measured using the diffraction spectrophotometer in the same energy range at RT. The onsets of spectral bands were estimated by applying standard methods based on the differentiation of the spectra which allow to find different critical points [43].

## Results

**Fig. 2:**
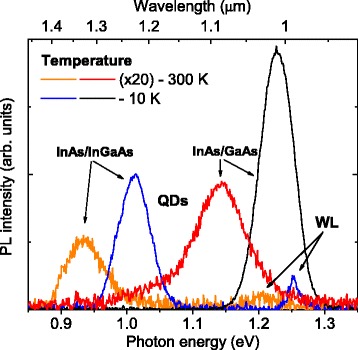
(*Color online*) Room temperature (*T* = 300 K) and 10 K PL spectra of the metamorphic InAs/In_0.15_Ga_0.85_As and conventional InAs/GaAs QD structures. The RT spectra have been multiplied by 20
B. Absorption
  Room temperature absorption spectra of the InAs/In_0.15_Ga_0.85_As and InAs/GaAs QD structures are presented in Fig. 3. The features of the metamorphic InAs/In_0.15_Ga_0.85_As QD structure spectrum in Fig. 3(a) have been identified elsewhere [30]. In particular, the threshold at 0.68 eV is related to the EL2 family defect centers [22–29]. The step at 0.85 eV is caused by the absorption in ensemble of the QDs, which corresponds to the onset of the QD peak in the PL spectrum at RT, whereas another one at 0.98 eV can be attributed to the transition from the first QD heavy hole excited state to the continuum of In_0.15_Ga_0.85_As MB conduction band states. The feature at 1.11 eV can be caused by the transitions between band and shallow defect levels in the MB, and the one at 1.17 eV can be related to the WL absorption, which corresponds to the WL PL peak at RT. But the rise after 1.17 eV is so sharp that can rather be attributed to the carrier generation in both the WL and In_0.15_Ga_0.85_As MB through shallow levels. The calculation of the In_0.15_Ga_0.85_As MB bandgap, considering the exact amount of strain for a thickness of 500 nm and the correct Varshni parameters, gives a value of 1.225 eV at 300 K [44].

A. Photoluminescence
  The PL spectra at both 10 and 300 K of the InAs QD embedded in the In_0.15_Ga_0.85_As MB and conventional InAs/GaAs QDs grown on *si*-GaAs substrates are presented in Fig. 2. The emission spectrum of the metamorphic QD structure at 10 K shows two bands at 1.02 (QDs) and 1.26 eV (WL), that redshift at RT to 0.94 and 1.20 eV, respectively, while InAs/GaAs QDs emit at 1.23 eV at 10 K and 1.15 at RT. It should be noted that the metamorphic QDs emit in the telecom range at 1.3 μm at RT as it have been described in earlier papers, while maintaining an efficient emission that is about only half of the emission intensity of InAs/GaAs QDs [4, 10, 15].

**Fig. 3:**
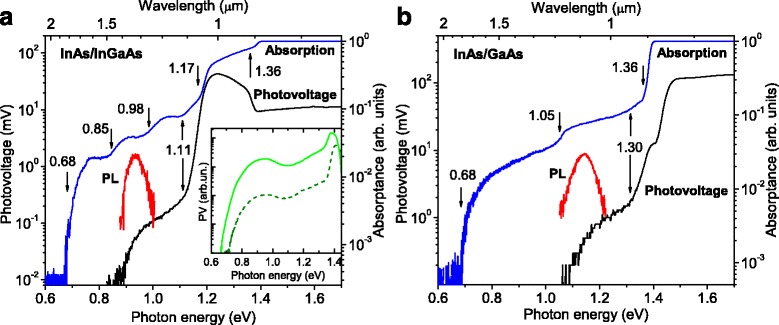
(*Color online*) Room temperature (*T* = 300 K) PV and absorption spectra of the **a** metamorphic InAs/In_0.15_Ga_0.85_As and **b** conventional InAs/GaAs QD structures. PV was measured contacted to only MBE layers; in the *inset*, the PV spectra measured through the semi-insulating GaAs substrates in the metamorphic and conventional QD structures (*solid and dash line*, respectively). The QD PL spectra in arbitrary units are shown for easier comparison
  Finally, the last step at 1.36 eV with the following plateau is obviously related to the absorption of the *n*-doped GaAs buffer layer, which might contain shallow levels, redshifting the interband absorption of the GaAs [24, 25]. In Fig. 3b, we show the absorption spectrum of the InAs/GaAs QD structure: the threshold of absorptance occurs at 0.68 eV, while a series of steps are observed at 1.05 and 1.3 eV, with the last step near 1.36 eV followed by plateau. The threshold and the last step occur at the same energies of the metamorphic structure allowing to attribute the first one to the EL2 family defect centers [22–29] and the 1.36 eV step to the GaAs buffer layers. The QD PL spectrum confirms the attribution of the 1.05-eV step to QD ground states, while the slight step at 1.3 eV can be attributed to the transitions in the WL [45].
C. Photovoltage and photocurrent
  The PV spectra of both the InAs/In_0.15_Ga_0.85_As and InAs/GaAs samples are also presented in Fig. 3. All transitions mentioned above in the absorption data contribute to the PV signal. However, no signal from the EL2 centers is observed in the spectra of samples contacted to the thick InGaAs or GaAs buffers. On the other hand, in structures where current flows also through the substrate, the PV signal onset occurs at 0.68 and 0.72 eV, for metamorphic and conventional structures, respectively (inset in Fig. 3). Additionally, the wide band peaked at 1.24 eV with edge at 1.11 eV is observed in the InAs/In_0.15_Ga_0.85_As sample PV spectrum, with fewer steps in comparison with the absorption spectrum (Fig. 3a). This PV spectrum feature points out at the correctness of assumption that after 1.11 eV, the carrier generation occurs mainly in the In_0.15_Ga_0.85_As MB including the way through shallow levels.

**Fig. 4:**
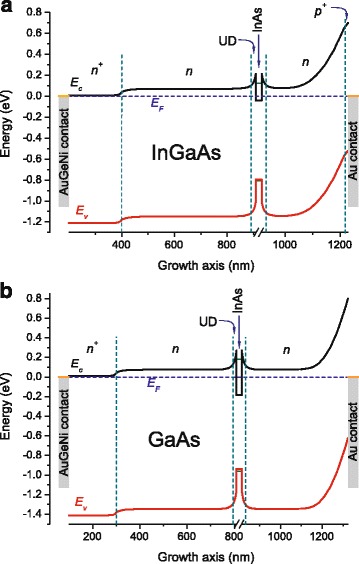
(*Color online*) Band profile of the **a** metamorphic InAs/In_0.15_Ga_0.85_As and **b** InAs/GaAs QD structures at 300 K calculated using Tibercad software

In PV measurements, the optically excited charge carriers drift to different sides according to the band structure slope. The Au Schottky barrier height is 0.7 eV for InGaAs and 0.8 eV for GaAs, resulting in a rather steep slope of energy bands at the surface of the top buffers [33, 34]. The band schemes of studied structures are pictured in Fig. 4 based on calculations using Tibercad software, as described above. Hence, the electrons (holes) excited in the buffers move to the sample bottom (top), thus giving a positive potential at the structure surface and a negative one at the AuGeNi contact. The wide space charge area at the Schottky barrier, about 200 nm with 0.7–0.8 eV height, makes difficult for electrons to move from QDs to the surface, so, at the existing conditions, electrons excited in the QDs and WL move predominantly towards the substrate side as well giving the same PV sign as the buffer layer. The sharp fall of the PV signal from the metamorphic QD sample above 1.36 eV is caused by an effect of the GaAs layers which has been described elsewhere [30]. The slight fall of the PV signal from the InAs/GaAs sample resulting in the small feature after 1.36 eV can be explained by the absorption edge of the MBE grown upper GaAs buffer, overshading the QDs and WL being obviously the more efficient contributors to PV. Then, the subsequent band-to-band absorption above 1.4 eV in the *n*-GaAs buffer leads to the sharp rise of PV.

**Fig. 5:**
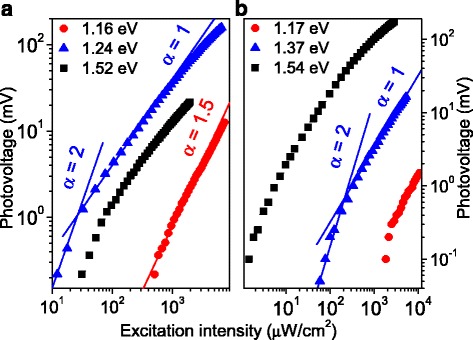
(*Color online*) Dependences of PV on the excitation intensity at different photon energies for **a** InAs/In_0.15_Ga_0.85_As and **b** InAs/GaAs structures; the *lines* are the functions *f*(*I*_ex_) ∝ (*I*_ex_)^α^

The dependences of PV on the excitation intensity (*I*_ex_) measured at characteristic points of the spectra are presented in Fig. 5. For the InAs/In_0.15_Ga_0.85_As sample (Fig. 5a), the point at 1.16 eV corresponds to the efficient absorption in the mentioned above shallow acceptors in the MB whereas the band-to-band absorption in the WL is too weak, 1.24 eV (spectrum peak) corresponds to the In_0.15_Ga_0.85_As band, while the value at 1.52 eV matches the plateau region. The near-surface absorption in the upper In_0.15_Ga_0.85_As MB predominantly occurs at so high energy; this can be concluded from the absorption spectrum. Indeed, according to absorptance data, only about 20% of light reach the GaAs layer at 1.36 eV. This part becomes certainly lower at higher energies.

The InAs/GaAs structure is studied at 1.17, 1.37, and 1.54 eV (Fig. 5b). The first point is in the range of efficient absorption in the QDs; the second one is the range where the transitions from the WL states are the most efficient whereas the absorption in the *n*-GaAs layer has no substantial impact. At the point of 1.54 eV, the signal is mainly caused by the intrinsic absorption in the GaAs buffer.

**Fig. 6:**
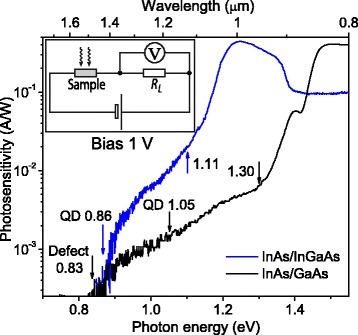
(*Color online*) Room temperature (*T* = 300 K) photosensitivity spectra of the metamorphic InAs/In_0.15_Ga_0.85_As and conventional InAs/GaAs QD structures. *Inset*: electric scheme of connecting the sample for PC measurements. *RL* Load resistance

The photocurrent has been investigated at 1 V negative bias (“−” at the top and “+” at the bottom contact). The measured spectra, presented in Fig. 6 as photosensitivity, show mainly the same structure features as the PV spectra considered above. The components of QDs, WL, and In_0.15_Ga_0.85_As or GaAs buffers as well as doped GaAs buffer layers manifest at the PC spectra at the same energies; however, the PC threshold of InAs/GaAs structure is observed at 0.83 eV that is much lower than the QD state transitions. It should be noted that this value coincides with that obtained by DLTS measurements for similar InAs/GaAs QD structure [15, 46, 47]. Measuring the DLTS spectra at different distances from QDs, 0.84 eV traps have been observed in the QD plane. Such traps have been attributed to defects related to the presence of ripened QDs [46]. This allows to consider 0.83 eV level as the InAs-QD/GaAs interface defect.

## Discussion

In the present work, we have focused on the observation of photoelectric properties of the metamorphic InAs/In_0.15_Ga_0.85_As QD nanostructures and conventional InAs/GaAs QD ones grown with similar design on thick buffers. The use of In_*x*_Ga_1 − *x*_As MB and embedding layers successfully shifts the QD optical transition energies to lower ones [4, 9, 15, 30], by reducing the QD strain [3–7]; as a side effect, MB-related defects are present in the InGaAs material. It should also be considered that a significant impact on the carrier transport and its lifetime may be caused by the formation of specific band bending at InGaAs/GaAs and InAs/InGaAs interfaces. Such features determine the properties of samples leading to differences not only in emission energies but also in PL emission intensity, photosensitivity, carrier leakage through defects, and other.

The PL characterization of the InAs QD-capped structures embedded in the In_0.15_Ga_0.85_As MB in comparison with the InAs QDs grown on GaAs has shown an efficient QD emission that is about a half of the emission of the InAs/GaAs QDs, known to be very efficient light sources [48, 49], at both 10 and 300 K (Fig. 2). At RT, the metamorphic QDs emit at 0.94 eV, while the InAs/GaAs ones emit at 1.15 eV. Considering that QD density of InAs/GaAs standard QDs is double that of metamorphic QDs [4], we can roughly estimate the efficiencies per one QD to be similar for both the samples. This evidences that the presence of the inherent InGaAs dislocations and defect levels do not severely impact the optical quality of the QD cladding layers [50].

It can be noticed that the investigated metamorphic QDs emit in the telecom range at 1.3 μm at RT, while maintaining a good PL efficiency that has been described in earlier papers [4, 9, 15, 30]. The following features of metamorphic structures affect on such PL redshift: (i) at the identical growth parameters, the metamorphic QDs self-assemble in the larger size and with the higher QD density [4]; (ii) the reduction of QD-cladding layer discontinuities downshifts the confinement levels by values of 0.01–0.1 eV (based on the calculation by Ref. [51, 52]); and (iii) some shift of confinement levels is caused by the strain reduction [4, 5].

The absorption and photoelectric characterization data agree with the experimental PL transitions in both structures. The spectra show the effective absorption of normal incident light in the QD, WL, and buffers (Fig. 3), overlapping in band positions with the PL data on the QD and WL transitions as taken from Fig. 2. All the transitions found in the absorption data contribute to the PV and PC signal.

Summing up the results, we can affirm that photoelectric properties are similar to the two types of structures. The QD components manifest in the photoelectric spectra as wide bands positioned at the same energies of PL ones. The main differences are due to the In_0.15_Ga_0.85_As MB which causes the reduction of the QD optical transition energies. On such structures, we still observe an effective detection of photocarriers coming from QDs. The photosensitivity spectra in Fig. 6 allow to quantitatively compare the structures under investigation. Being comparable from buffers (near 0.4 A/W), the photosensitivity of the metamorphic InGaAs nanostructure is higher in the QD range, 4 × 10^−4^ vs 2.7 × 10^−4^ A/W. Considering that QD density of InAs/GaAs standard QDs is double that of metamorphic QDs [4], it appears that PC excited in metamorphic QDs is a more efficient process. Taking into account that the PL efficiencies per one QD of both the structures are similar, one can conclude that an additional leakage of photogenerated carriers might occur in the InAs/GaAs sample by means of the non-radiative recombination through the interface levels like energy of conductivity band (*E*_*c*_)—0.83 eV reported earlier [15, 47] and found by PC. It is noteworthy that a more comprehensive comparison is possible in PC, as the generation and recombination of carriers are involved in the PC mechanism, along with their transport and loss through non-radiative recombination in buffer.

The level *E*_*c*_—0.83 eV was not observed in the InAs/In_0.15_Ga_0.85_As samples (Fig. 6): in previous works on DLTS, such a level was attributed to extended defects related to strain relaxation in the vicinity of QDs [46]. Hence, present results suggest that such levels might not be present in metamorphic structures, possibly due to the lower level of strain between InAs QDs and InGaAs layers, in comparison with the InAs/GaAs system.

On the other hand, an indirect evidence of some recombination center existence has been found in both the structures from the PV dependences on excitation intensity. All the PV dependences tend to be superlinear close to (*I*_ex_)^2^ at lower intensities. Since such a dependence can be explained within a model of Shockley-Read recombination of free carriers through two types of centers [48, 49], this could indicate the presence of defects in both GaAs and InGaAs buffers. For higher excitation intensities, the defect states are saturated, and henceforth, the curves, except at 1.16 eV for metamorphic sample, have a linear part in the middle which transforms to sublinear for the highest intensities. Such a behavior might be explained by direct recombination of the nonequilibrium carriers in a doped semiconductor, considering simple rate equations [53, 54]. In contrast, the dependence at 1.16 eV of the metamorphic sample in Fig. 5a is close to (*I*_ex_)^3/2^. This shape also implies recombination of carriers excited band-to-band, i.e., within QDs, through at least two recombination levels [50]. We think this process occurs through shallow levels, possibly related to defects located in MBE grown buffers and strained interfaces that account for the 1.11 eV sharp feature in the absorption and PV spectral curves. Those defects are commonly observed by means of DLTS [15, 24, 25] and TSC [26, 27, 29] measurements.

As for EL2 defect levels in buffer layers located near 0.75 eV below the conduction band [22–25], no signal from these centers is observed in the spectra of samples with the contacts on the thick InGaAs or GaAs MBE buffers, while measuring PV through the substrate, the signal onset occurs at 0.68 and 0.72 eV for metamorphic and standard structures, respectively (inset in Fig. 3). As well the onset of both the absorption spectra at 0.68 eV, we attribute to the transition from EL2 defect center to the conduction band considering the possible way through the shallow defect levels [24–27, 29]. Similar redshift of EL2-related bands in optical transition have been described for InGaAs/GaAs nanostructures [27, 30]. However, the fact that no signal below QD spectral band is observed in the PV spectra from only MBE layers means a low amount of EL2-like non-radiative recombination centers in the metamorphic and GaAs buffers overall, providing the effective detection of photocarriers coming from even a single layer of QDs, whereas the high density of recombination centers in the GaAs substrates and *n* ^+^-GaAs layers of the structures under investigation has been confirmed by the PV characterization of samples measured through the substrate (inset in Fig. 3).

Hence, by studying samples with different contact configuration (only on MBE layers or on the bottom part of the substrate), we were able to conclude that the regions with higher defect density are the GaAs substrate and GaAs buffer layers [15, 22, 23, 55, 56]. Henceforth, the InGaAs/GaAs interface region did not show to have a strong influence on the electrical properties of the QD structures.

## Conclusions

We can conclude that the photovoltaic properties of both types of studied samples (i.e., metamorphic InAs/InGaAs and pseudomorphic InAs/GaAs QD structures) are mostly similar. An efficient detection of photocarriers has been shown for the sample with InGaAs MB, which is instrumental to redshift the QD emission towards the telecommunication range (1.3 μm). A critical influence of the EL2 deep levels on the photoelectrical properties of both the structures was found when the current flowed through the substrate, while no such effect was observed in the PV spectra in the case of samples contacted to the MBE layers. However, the slight recombination of the photoexcited carriers through non-radiative centers was observed in intensity-driven PV measurements of both samples. The latter contact geometry, along with the thick buffers on substrate, allowed for strong suppression of the influence of photoactive deep levels in the interfaces and *si*-GaAs substrate on the photoelectric properties of both structures. No relevant contribution of a level at *E*_*c*_ *—*0.83 eV was observed in the metamorphic InAs/InGaAs QD structures, in stark difference with the InAs/GaAs ones. This result allows us to conclude that the metamorphic InAs/InGaAs heterostructures are not affected by levels related to extended defects in the vicinity of QD, apparently due to a lower strain in comparison with the InAs/GaAs structure. Based on the results on optical and electrical properties of metamorphic QDs presented here, it can be concluded that efficient photonic devices at 1.3 μm can be developed with similar nanostructures as an active material. This could be of great interest for the fabrication of GaAs-based photonic systems operating in the telecom range.

## Abbreviations

AFM: Atomic force microscopy
ALMBE: Atomic layer molecular beam epitaxy
DLTS: Deep-level transient spectroscopy
*E*_*c*_: Energy of conductivity band
*I*_ex_: Excitation intensity
MB: Metamorphic buffer
MBE: Molecular beam epitaxy
PC: Photoconductivity
PL: Photoluminescence
PV: Photovoltage
QD: Quantum dot
RT: Room temperature
*si*: Semi-insulating
TSC: Thermally stimulated conductivity
WL: Wetting layer
